# Connectivity, Coverage and Placement in Wireless Sensor Networks

**DOI:** 10.3390/s91007664

**Published:** 2009-09-28

**Authors:** Ji Li, Lachlan L.H. Andrew, Chuan Heng Foh, Moshe Zukerman, Hsiao-Hwa Chen

**Affiliations:** 1 ARC Special Research Centre for Ultra-Broadband Information Networks (CUBIN), University of Melbourne, Parkville 3010, Australia; E-Mail: j.li@ee.unimelb.edu.au; 2 Centre for Advanced Internet Architectures (CAIA), Swinburne University of Technology, Melbourne, Australia; E-Mail: landrew@swin.edu.au; 3 School of Computer Engineering, Nanyang Technological University, 639798 Singapore; 4 Department of Electronic Engineering, City University of Hong Kong, Kowloon, Hong Kong; E-Mail: m.zu@cityu.edu.hk; 5 Department of Engineering Science, National Cheng Kung University, Taiwan; E-Mail: hshwchen@ieee.org

**Keywords:** wireless sensor networks, connectivity, coverage, relay placement, percolation theory

## Abstract

Wireless communication between sensors allows the formation of flexible sensor networks, which can be deployed rapidly over wide or inaccessible areas. However, the need to gather data from all sensors in the network imposes constraints on the distances between sensors. This survey describes the state of the art in techniques for determining the minimum density and optimal locations of relay nodes and ordinary sensors to ensure connectivity, subject to various degrees of uncertainty in the locations of the nodes.

## 1. Introduction

This survey provides an overview of wireless sensor network (WSN) connectivity, and discusses existing work that focuses on the connectivity issues in WSNs. In particular, we are interested in maintaining connected WSNs and their connectivity related characteristics including sensor node placement, as well as the construction of a small connected relay set in WSNs. We aim to review extensively the existing results related to these topics, and stimulate new research.

Sensor networks have a long history, which can be traced back as far as the 1950's. It is recognized that the first obvious sensor network was the Sound Surveillance System (SOSUS) [1, 2]. The SOSUS was made up of an array of acoustic sensors that were interconnected by wired cables and were deployed by the US in deep ocean basins during the Cold War to detect and track Soviet submarines. In its early stages, the development of sensor networks was mainly driven by military use, in which sensor nodes were wired together to provide battlefield surveillance.

Evolution of technologies has driven sensor networks away from their original appearance. With the emergence of integrated sensors embedded with wireless capability, most of current sensor networks consist of a collection of wirelessly interconnected sensors, each of which is embedded with sensing, computing and communication components. These sensors can observe and respond to phenomena in the physical environment [3]. Such sensor networks are referred to as wireless sensor networks (WSNs). These WSNs provide flexibility in deployment and maintenance, exploit the ability of wireless networks to be deployed in highly dynamic environments and hence enable sensor networks to be potentially used in a wide range of civilian and military applications, including security surveillance (e.g., to alert of terrorist threats), environmental monitoring, habitat monitoring, hazard and disaster monitoring and relief operations, health field applications, and home applications (e.g., smart environments) [3]. The wireless communication in WSNs can be either ad hoc (multi-hop) or single-hop wireless transmission [4]. Though the latter is popular in short-range applications, such as smart homes, the former, ad hoc technique, attracts more interests due to its high flexibility and ability to support long-range, large scale, and highly distributed applications. In this survey, we only focus on wireless sensor networks adopting multi-hop transmission.

In a WSN, after collecting information from the environment, sensors need to transmit aggregated data to gateways or information collection nodes. It is important to ensure that every sensor can communicate with the gateways. Due to the multi-hop communication of WSNs, a sufficient condition for reliable information transmission is full connectivity of the network. A network is said to be fully connected if every pair of nodes can communicate with each other, either directly or via intermediate relay nodes. Due to the large number of sensors in a WSN, the total cost could be high for the whole network, though the cost of each individual sensor is low. Therefore, it is important to find the minimum number of nodes required for a WSN to achieve connectivity.

Another related important problem for WSNs is finding a small connected relay set to assist in routing. Multi-hop WSNs need to perform efficient routing. Since mobile ad hoc networks (MANETs) and WSNs often have very limited, or even does not have, fixed infrastructure, the routing process in such networks is often complicated and inefficient; it can generate a large amount of overhead, and there are many possible paths, due to the broadcast nature of the wireless communications. Thus it is helpful to find a small connected set of sensor nodes to form a routing “backbone”, and restricted all other nodes to connecting to this backbone by a single hop. This node set can also help to resolve the broadcast storm problem [5], which is often caused by blind flooding.

As WSNs may be deployed in inaccessible terrains, and may contain a tremendous number of sensor nodes, it is often difficult or impossible to replace or recharge their batteries. Thus, energy conservation is critical for WSNs, both for each sensor node and the entire network level operations. Various approaches have been proposed to reduce energy consumption for sensor networks. For example, for the network level operations such as routing, if only a small fraction of sensors are involved in the routing process, the rest of the sensors can be turned off to save energy. This scheme is supported by the hardware and software advances that leverage the capability of temporarily shutting down those sensors that are not involved in any network operations. For instance, Rockwell's WINS sensor nodes can achieve a factor of ten power reduction by shutting down the radio transceiver, compared to those idle nodes whose transceivers are on [6]. However, a prerequisite for this type of energy saving scheme is that the WSNs still perform all the required functions even with some nodes turned off. This raises an important research problem: what is the maximum number of sensors that can be turned off, while maintaining functionality of the WSN? This problem is equivalent to minimizing the total number of active nodes, subject to ordinary operations of the system. The selected sensors will function as backbone relay nodes to maintain communications within the entire sensor network. A further important problem, which is beyond the scope of this survey, is how to optimally shut off and turn on sensors over time to maximise network lifetime [7].

The remainder of this paper is organized as follows. Section 2 gives a brief introduction to the graph models applied to wireless network investigations. Section 3 provides an overview of the prior results for connectivity studies in wireless ad hoc networks and WSNs, including percolation theory. Section 4 describes models with more general radio coverage patterns, and some hybrid models. The implications of connectivity on the achievable capacity are discussed in Section 5 Section 6 considers the construction of a small connected relay set, such that the packet delivery can be achieved by forwarding packets using only sensors in the relay set. Section 7 covers the optimal placement of sensor nodes, which has a fundamental impact on the connectivity and other operational requirements of WSNs. Section 8 summarizes this survey.

## 2. Graph Models

Connectivity is critical for WSNs, as information collected needs to be sent to data collection or processing centres. This is only possible if there is a path from each node to that collection centre. The connectivity of a WSN is usually studied by considering a graph associated with that WSN.

A WSN or a wireless ad hoc network is often represented by a graph in which vertices correspond to the communication nodes, and a directed edge from one vertex to another indicates that the node corresponding to the former can send data directly to the node corresponding to the latter. It is common to assume that propagation conditions can be modelled simply by there being a “transmission range” within which transmission is possible, and outside of which it is impossible. If all nodes have equal transmission ranges, then the graph becomes undirected.

A network is called connected if this associated graph is connected. A graph *G* is connected if and only if there exists a path between any pair of its vertices [8]. If a network is connected then any pair of nodes can communicate with each other, possibly taking multiple hops through relay nodes. It is sometimes useful to consider stronger forms of connectivity, such as *k*-connectivity, in which the network remains connected even if *k –* 1 nodes are removed. If a network is *k*-connected (*k* ≥ 2), it has better fault-tolerance than if it is merely 1-connected. Ensuring *k*-connectivity extends the network lifetime if nodes fail at random times.

Weaker notions of connectivity are also possible, such as the requirement that each node need only be connected to one of a set of base stations [9]. This paper will focus on the intermediate case in which the entire network is required to be 1-connected.

It is clear that the connectivity of a WSN is related to the positions of nodes, and those positions are heavily affected by the method of sensor deployment. In general, there are two types of approaches to deploy sensors in a WSN: deterministic deployment, where sensors are placed exactly at pre-engineered positions, and the random deployment, where nodes are deployed at random positions. For the deterministic deployment, networks are carefully planned, and nodes are placed at desired positions. If specifications of nodes are known, it is not difficult to determine whether the network is connected, and if not, to add relay nodes where needed. Although deterministic deployment has many advantages, in order to reduce installation costs, it has often been proposed that large WSNs which contains very large numbers of nodes be deployed randomly. Nodes may be dispersed by a moving vehicle or artillery shell [3]. Therefore, sensors often have nondeterministic positions, and the analysis of connectivity of such a type of networks involves the modelling of random networks.

Random graphs are often applied to model communication networks to highlight their randomness. Mathematically, a random graph is a graph that is generated by a stochastic process [10]. The theory of random graphs began with Erdos and Renyi's pioneer work in the late 1950s and early 1960s, which considered a class of random graphs now called Erdős-Rényi graphs [11, 12]. An Erdős-Rényi graph *G*(*n, p*) is a graph with *n* vertices, in which each of the ${\text{C}}_{n}^{2}$ possible edges exists with probability *p*, independent of all other edges. This class was originally used as a tool in the field of combinatorial mathematics. However, it is now widely accepted as a model for many fields, such as communications [13–15], social studies [16, 17] and biology [18]. Independent of Erdős and Rényi, Gilbert [19], Austin *et al.* [20], and Ford and Uhlenbeck [21] also studied the statistical aspects of graphs. However, unlike Erdős and Rényi, they used enumeration methods, which are essentially deterministic, to estimate exact values of graph properties, while Erdős and Rényi studied the distributions of graph properties. Thereby, Erdős and Rényi are often credited with the foundation of the theory of random graphs.

As the probability of an edge existing between each pair of nodes is equal in an Erdős-Rényi graph, this model is not well suited to WSNs, which are embedded in two (or three) dimensional space, and in which the probability of a link existing is very much higher between nodes which are geometrically close. Moreover, as discussed by Chlamtac and Faraó [22], Erdős-Rényi graphs do not consider correlations between different links. Therefore, we need new methods to model networks with randomness.

A natural candidate for random network modelling is the class of *Random Geometric Graphs* [23]. With node set *V*, a *geometric graph G* = (*V, r*) is equivalent to a graph *G*_1_ = (*V, E*), in which the vertex set *V* is embedded in a metric space, and *E* = {(*u,v*)|∀*u,v* ∈ *V*,‖*u*− *v*‖ ≤ *r*}. If the nodes in *V* are independent and identically distributed (i.i.d.) and uniformly distributed in the metric space, *G* is called a random geometric graph. In this case, the locations of nodes form a Poisson process if the number of nodes is Poisson distributed, or infinite in the case of an unbounded metric space.

**Figure 1.**
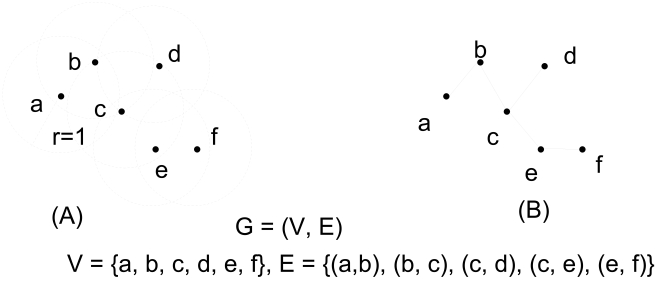
An example of unit disk graphs.

An important special case of geometric graphs is the class of unit disk graphs. A unit disk graph is a graph embedded in Euclidean space that has an edge between any two vertices whose Euclidean distance is less than 1. If the positions of vertices of the unit disk graph are random, this unit disk graph is a random graph, and it is a random geometric graph if the locations of the vertices are i.i.d. and uniformly distributed. Figure 1 shows an example of the construction of a unit disk graph. As can be seen, any pair of nodes that have a distance less than or equal to *r* = 1 are connected by an edge.

Gilbert [24] studied unit disk graphs on an infinite plane, under the name *Random Plane Networks.* He appears to be the first to suggest their applicability to connectivity studies of packet radio networks.

## 3. Connectivity in Wireless Ad Hoc and Sensor Networks

One of the most interesting questions regarding the connectivity of random WSNs concerns finding limiting regimes for which the connectivity becomes almost sure to occur. Typically these regimes involve the number of nodes becoming large.

Among the most celebrated results is that of Gupta and Kumar [25]. This showed that for *n* nodes uniformly i.i.d. in a disk of area *A*, with the particular radio transmission range

(1) $$r(n)=\sqrt{\left(A(\log n+\gamma_{n}))/(\pi n\right)},$$

the network is asymptotically almost surely connected if and only if *γ_n_* → +∞, by using percolation theory. The importance of this result was shown in [26], where they used it to show that the capacity per user of a static ad hoc network decreases as $1/\sqrt{n}$ as the number of nodes increases, under several models of single-user communication. Note however that sophisticated methods such as proposed by Ozgur, Leveque, and Tse [27] can circumvent this limit.

Before returning to this type of result, let us review some results for infinite networks

### 3.1. Percolation in Infinite Graphs

Many of the results concerning connectivity of large ad hoc networks are derived from results in percolation theory This section gives an introduction to percolation theory, and reviews some existing work related to the network connectivity studies. An introduction to theoretical aspects of percolation can be found in [28] and [29].

The notion of percolation was developed by Broadbent [30], Broadbent and Hammersley [31], and Hammersley [32] to mathematically model the phenomenon of fluid flowing through random porous materials, e.g., water soaking a sponge. The condition of holes in the material being sufficiently connected that the entire sponge becomes wet is analogous to long-range communication being possible in a multi-hop network. An important characteristic of percolation models is the existence of phase transitions. The degree of connectedness is typically controlled by some parameter, such as the radio transmission range, and there is a critical value below which an infinite network is very fragmented, and above which it is “partially connected”, in a sense which will be described below.

Mathematically, percolation theory studies infinite random graphs, which contain an infinite set of vertices *V* spreading over an infinite plane. Correspondingly, percolation theory in wireless ad hoc networking areas focuses on the connectivity of infinite networks, which contain an infinite number of nodes that are distributed on an infinite plane. In an infinite plane, the probability that at least one node is isolated typically tends to 1, and thus full connectivity defined previously almost never occurs. Therefore, the literature often consider the formation of a connected component with an infinite number of nodes. This is called partial connectivity, as distinct from full connectivity, because it cannot guarantee that all nodes are connected. An infinite connected component is a collection of vertices, which is an infinite subset *V*_1_ of *V*, such that there is at least one path between any arbitrary pair of nodes in *V*_1_. An infinite connected component corresponds to a communication network of infinitely many nodes spread over a large geographic plane. Therefore, the existence of an infinite connected component implies the capability of long distance communication via multi-hop links.

Early studies of percolation constrained vertices to lie on a regular lattice. This requirement was relaxed by Gilbert [24], who introduced continuum percolation in which vertices can lie anywhere in a continuum such as a plane. This seminal work indicated its applicability to multi-hop communications. It investigated P(*n*), the probability that there exists a connected component containing *n* nodes; in particular, this was studied as *n* goes to infinity, giving the probability of forming an infinite connected component. It was expressed as a function of average nodal degree *E* = *λπr*^2^, where *λ* is the density of nodes. It was shown that there is a critical value *E_c_* of *E*, such that an infinite connected component is only possible when *E* > *E_c_*. Although the exact value of *E_c_* was not known, [24] showed that 1.75 ≤ *E_c_* ≤ 17.4. Philips *et al.* [33] improved the results by providing a tighter bound of *E_c_* as 2.195 < *E_c_* < 10.526. Besides the analytical approaches to evaluating *E_c_*, numerical methods [34] have also been used to obtain bounds for *E_c_*. These results provide some necessary conditions to construct a connected network in a large plane. As percolation theory focuses on the infinite networks and studies the existence probability of an infinite connected component, the connectivity it emphasized is indeed partial connectivity rather than full connectivity. However, as the density increases, the network becomes fully connected in the sense that the probability of a random node not being in the infinite connected component tends to 0.

Designing a large network to achieve full connectivity is much more demanding than designing to ensure that a large percentage of nodes are connected, with an associated increase in energy requirements. This is quantified by simulation in [35] and [36] and analytically in [37]. The largest connected component of a network is called the giant component. In the context of wireless ad hoc networks, the size of the giant component has been evaluated under a wide range of assumptions [36]. Specifically, analytical bounds are known [37] for the minimum transmission range to achieve a giant component that contains more than a given number of nodes.

Percolation theory can also be applied to 1-dimensional (1-D) networks, such as sensor networks following rivers. Percolation is much more limited in 1-D, since there is much less variety of possible paths than in higher dimensions. However, some fundamental results are known. For example, the asymptotic connectivity probability of a 1-D network exhibits a strong zero-one law even if the locations of all the nodes independently follow an arbitrary identical distribution, as long as this distribution admits a non-vanishing density function [38]. The zero-one law indicates that the maximal spacing between adjacent nodes converges in probability to a scaling which is dependent on the total number of nodes *n*, and the density function *f*, which all the nodes follow. Note that this result is important as it provides a deterministic estimate of the critical transmission range. The application of percolation to the connectivity of 1-D networks has been surveyed by Han and Makowski [39].

### 3.2. Connectivity on Finite Graphs

The effect of a phase transition to partial connectivity in infinite networks is mirrored by a similar effect for complete connectivity in large but finite networks.

Philips *et al.* [33] considered a geometric random graph model of a wireless packet radio network with a Poisson process of nodes within a finite but large area *A*. They extended Gilbert's results by studying the relationship between *A* and the average nodal degree *E* to ensure that all nodes in the network are connected. It was proved that for any *ε* > 0, with transmission range *r* given by $r=\sqrt{(1-\varepsilon )\log A/\pi \lambda }$, the probability of being connected tends to 0 as *A* becomes large. It was also conjectured that given $r=\sqrt{(1+\varepsilon )\log A/\pi \lambda }$, *ε* > 0, the network is almost surely connected. This conjecture would imply that to obtain asymptotic almost surely connectivity, the expected number of nodes within transmission range of an arbitrary node should grow logarithmically with network area. This result is formulated with the density fixed and the area growing; for comparison with (1), the radius required for a fixed area with increasing *n* = *λA* would be

(2) $$r(n)=\sqrt{\left(A(\log n+\varepsilon \log n))/(\pi n\right)}.$$

It can be seen that this conjecture, in two dimensions, is established and tightened by (1), which replaces *ε* log *n* by an arbitrary function of *n* that tends to infinity.

Piret [40] studied Philips *et al.*'s problem for 1-D networks, and showed that the conjecture in [33] does not hold in that case. There are many cases in which 1-D networks are an exception, with more stringent connectivity requirements.

So far, we have considered the problem of determining the radius required to ensure a high probability of connectivity in large networks. The converse problem of estimating the probability of connectivity for a given radius is if considerable engineering interest.

Consider nodes placed uniformly in a square region, with a pair of nodes connected if their distance is below a threshold, *r*_0_. As the number of nodes becomes large, the probability that the network is connected (or *k*-connected) is asymptotically the same as the probability that no node is isolated (or has degree less than *k*). This follows from the fundamental result in random graph theory that, for random geometric graphs of a hypercube of two or more dimensions: the minimum connectivity radius for achieving *k*-connectivity is exactly equal to the minimum radius for achieving a minimum nodal degree *k*, with probability tending to 1 as the number of vertices goes to infinity [41]. This probability can be calculated by treating the node locations as a 2-D Poisson process [42]. This result holds for more general regions than squares, although not to all 2-D regions. Note that the proof in [41] (and hence the corollary in [42]) only applies to the square case. The result also does not establish almost sure connectivity for finite graphs, as suggested in [42].

The analogous problem of finding the probability of a finite 1-D network being connected can be addressed directly. Considering a 1-D interval with *n* nodes uniformly distributed within it, Desai and Manjunath [43] and Gore [44, 45] studied the probability that the network is connected for a given radio range. Foh and Lee [46] enhanced the results by providing a closed form expression for the connectivity probability of a 1-D WSN consisting of two additional border nodes in the 1-D interval.

In a practical sensor deployment, it may not be realistic to assume that the nodes are identically distributed. An alternative model, studied in [47], is for the nodes to have target locations spaced uniformly, but for the true locations to have identically distributed offsets from those targets. This study quantifies how the uncertainty in the offsets increases the number of nodes which need to be deployed. This approach requires substantially fewer nodes than simple identical distribution of sensors, with concomitant cost savings.

### 3.3. Techniques to Improve Connectivity

As connectivity is so important to function a sensor network, some techniques have been proposed to improve the connectivity by extending the transmission range of sensors. These techniques include cooperative transmission [9, 48], and the application of directional antennas [49].

Cooperative transmission is an exploitation of distributed beam-forming. With cooperative transmission, it is possible to accumulate the transmission power from different nodes to achieve a higher power to transmit identical information, and hence the transmission range can be greatly enlarged, and the connectivity of the whole network can be improved. To implement cooperative transmission, all nodes that are transmitting the same message should synchronize their transmission and superimpose the emitted waveforms on the physical medium, so that the power can be summed up to help the detection at the receiver side. This type of techniques are especially helpful to eliminate the nodes isolation and network separation for large and sparse networks. Krohn *et al.* [9] built a prototype platform to demonstrate that using cooperative transmission can actually increase the network connectivity.

Another technique with renewed interests in the network connectivity area is the use of directional antennas [49]. It was proposed to equip wireless devices with directional antennas, which can focus transmission energy in one or two directions, as a large portion of transmission energy from an omnidirectional antenna is wasted in the directions without receivers. Directional antenna can greatly reduce the transmission power while maintaining the same level of connectivity Accordingly, the sensors with directional antennas can transmit to a receiver with longer distance if the transmission power remains the same. Kranakis *et al.* [49] showed that when highly-directional antennas are applied to sensors, significant savings on the energy can be achieved, compared to that with omnidirectional antennas. This result indicated that under the same energy consumption constraints, sensors with directional antenna nodes can reach longer, and achieve better connectivity. In [49], the authors also derived the theoretical bounds on the maximum beam width to achieve the energy saving. Examples of applications of directional antennas include [50], and references therein.

Given the complexity of these two techniques on one hand, and the requirements of simple design of sensors on the other, clearly the challenge of improving connectivity in sensor networks still exists.

## 4. Less Regular Connectivity Models

Geometric random graphs, such as the unit disk model, are tractable because of the high degree of symmetry they assume. In real networks, the coverage regions of different nodes have different areas, and are highly non-circular. Many more realistic models have been studied in the context of both percolation and connectivity of finite graphs.

### 4.1. Non-uniform Disk Radii

If each node can adjust its transmission power independently, they have inhomogeneous transmission ranges. This model has also received considerable attention. Xue and Kumar [51] showed that if inhomogeneous ranges are allowed, asymptotic connectivity is maintained if each node in a random network with *n* uniformly distributed nodes connects to Θ(log *n*) nearest neighbors. They further showed that the constant in the asymptotic relationship is greater than 0.074 and less than 5.1774, i.e., the network is almost surely disconnected if the asymptotic nodal degree *k* < 0.074 log(*n*), and the network is almost surely connected if *k* > 5.1774log(*n*). The range of the constant *k* is further reduced to the interval [0.3043,0.5139] [52]. The exact value of the critical constant remains an open problem.

Having inhomogeneous transmission ranges causes the corresponding graph to be directed. This is not desirable from an engineering point of view, both because it complicates routing, and also because it bars the use of link-layer acknowledgements and retransmission. The solution taken by Blough *et al.* [53] is to consider connectivity induced by only the symmetric edges; that is, two nodes are connected only if their separation is less than the minimum of their transmission ranges. By applying Xue and Kumar's results, Blough *et al.* developed a topology control mechanism that generates a network topology by connecting each node to its *k*-nearest neighbors. The value of *k* that can provide high probability of connectivity was numerically estimated to be around 9 for networks of 50–500 nodes. Other work that obtains the radio transmission ranges can be found in [54–57].

### 4.2. SINR Model

The concept of “transmission range” used in most connectivity studies is a marked over-simplification. In real networks, the presence of a link depends on both the signal strength and the background interference. One of the main drawbacks of disk-based models is that they do not consider interference, though dense networks produce strong interference.

To take interference into account, Dousse *et al.* [58] used another model, the signal-to-interference-plus-noise-ratio (SINR) model, in which two nodes are connected if SINR between the transmitter and receiver exceeds a certain threshold. As signal strength is considered in this model, the connectivity of the network depends on the selection of the attenuation function. It was proved that percolation occurs for the SINR model in a similar way as in the disk models. It was also shown that the commonly used power law attenuation function results in symmetry properties, and attenuation functions with finite support (i.e., a finite transmission range) can also achieve percolation. The authors showed that an efficient CDMA system with small orthogonality factors can improve the connectivity. However, due to the difficulty of designing efficient CDMA codes to achieve a small orthogonality factor, a simple TDMA scheme was proposed, which also percolates.

### 4.3. Shadowing

In real networks, obstacles block the path of the signal, and cause random anisotropic signal strengths. This effect, called *shadowing*, means that some links may exist to quite distant nodes, while there may be no links to quite close nodes.

The most widely accepted model of shadowing is log-normal shadowing [59]. This adds a random, normally distributed component to the path loss expressed in decibels, or equivalently multiplies the path loss by a log-normal random variable. In [60], it was shown that the optimal transmission power in a spread spectrum system should be selected such that the average number of nodes closer to the transmitter than the receiver is *ϕ*(*G/b*)^2/^*^ζ^*, where *ϕ* is a constant depending on the physical systems, *G* represents the spread spectrum processing gain, *b* is the threshold for the outage signal to noise ratio and *ζ* is the power loss factor.

Bettstetter and Hartmann [61] studied the probability that a single node is isolated in a log-normal shadowing environment with Poisson distributed nodes. A tight bound for the Poisson density to achieve a highly connected subnetwork is derived. It was shown that the fading would influence the connectivity significantly

Other models of shadowing are also possible. An interesting result arises if we consider the coverage area of each node to be a deterministic but irregular region, rather than a disk. Booth *et al.* [62] show that this irregularity enables the network to percolate in a lower density if the footprint of each node is convex, which indicates that sufficient conditions for connectivity obtained by disk-based models hold in general for unreliable and non-rotational symmetric wireless channels. However, for networks that each node produces arbitrary footprint, the connectivity is still an open problem.

### 4.4. Hybrid Models including Wired Infrastructure

Driven in part by results showing that the capacity per node decreases in large networks [26], many researchers have considered hybrid networks which contain not only peer nodes (sensors), but also some wired base stations, which can communicate to sensors via single or multi hops. Percolation in a hybrid network has also been studied. Booth *et al.* [63] and Dousse *et al.* [64] studied algorithms for base station placement to cover Poisson distributed nodes and the impact of base stations on the connectivity in two-dimensional sparse networks respectively. The results suggest that base stations help increasing the network connectivity significantly, when the node density in one dimension is much higher than the density in the other dimension.

## 5. Relationship between Connectivity and Capacity

Network capacity or throughput is an important constraint when considering the connectivity of ad hoc networks. In a wireless ad hoc network, the increase of the transmission power could increase the transmission distance of each node, which leads to the increased probability of network connectivity. However, the large power results in severe interference within the network, which reduces the network capacity and degrades the performance of decoding at receivers. On the other hand, reducing the transmission range by reducing the transmission power can limit the interferences, but reduces the probability of connectivity and increases the number of hops required to reach the destination. Therefore, the tradeoff between connectivity and network capacity has been widely studied.

### 5.1. Capacity Constraints imposed by Connectivity

As mentioned previously, the work of Gupta and Kuma [26] pointed out that the minimum density required for connectivity limits the possible throughput per node. They considered both a SINR model and model based directly on distances, for both optimal and random placement of nodes. For systems with *n* nodes, they established an upper bound on the capacity under the SINR model of $\Omega (1/\sqrt{n})$ in each case, and lower bounds of $\Omega (1/\sqrt{n})$ for optimal placement and $\Omega (1/\sqrt{n\log n})$ for random placement. Franceschetti *et al.* [65] showed that the lower bound for random placement is also $\Omega (1/\sqrt{n})$, giving matching asymptotic upper and lower bounds.

The fact that the capacity scales less than linearly with the number of nodes has prompted the study of hybrid networks containing some wired base stations. However, it has been shown that a linear scaling of capacity with the number of nodes is possible if the modeling assumptions are relaxed.

As already mentioned, Ozgur, Leveque, and Tse [27] showed that hierarchical cooperative beam-forming can, in principle, achieve capacity proportional to the number of nodes, albeit at the expense of requiring an unrealistic degree of cooperation.

Dousse *et al.* [66] demonstrated that the linear scaling is possible if a fixed (but arbitrarily small) fraction of the nodes are allowed not to be connected. Naturally, the achievable rate per node is a function of the fraction of nodes which are allowed to remain disconnected from the main connected component. In particular, if we want to impose a minimum data rate of transmission between nodes, then as the number of nodes goes to infinity, a fraction of nodes, which is a function of the required data rate, are automatically disconnected. This result was proved both for a Boolean (disk) model, and for a novel capacity-based model. In the latter model, the network is considered connected if and only any pair of nodes can communicate with a predefined minimum data rate, using other nodes as relays.

Note that the connectivity requirement is only an issue if the designer has control over the transmission range, but cannot add wireless relay nodes. An often overlooked consideration in these studies is that the *total* network capacity increases as the number of nodes increases. The fact that the capacity per node decreases as more nodes are added arises because it is assumed that additional nodes also have data to carry. If additional relay nodes are introduced purely to provide connectivity, then these nodes have no data of their own to send, and so do not reduce the capacity available for the original users. To see this, consider a network of a finite density of user nodes. Superimpose on this (a) a very much higher density of ad hoc relay nodes and (b) a planar wired network. In the ad hoc network, users can communicate by relaying data over the relay nodes lying on the paths of the wired network. As the density of the ad hoc network increases, the required transmission ranges vanish and so the interference between “virtual wires” also vanishes. This allows the users to communicate with arbitrarily high capacities.

To investigate the relationship between connectivity and capacity, Dousse and Thiran [67] proved that the attenuation function is one of the major factors affecting the properties of dense ad hoc networks. Under the assumption of power law attenuation, the network connectivity probability becomes higher as the density of nodes increases, even though the interference also increases. The increase of network total capacity is bounded by $\sqrt{n}$. However, the total network capacity remains constant with attenuation functions that are bounded above, and the network maintains connectivity at the expense of capacity.

### 5.2. Improving Capacity by Denser Connectivity

Another approach has been taken to studying the capacity of sensor networks, in the classic work [68] presented in 1978 by Kleinrock and Silvester, which sought to maximise throughput in networks using the ALOHA medium access control scheme. They considered a packet radio network with Poisson distributed terminals and homogeneous transmission ranges. In their model, the one-hop progress of a packet in the direction from a source to a destination is maximised by setting the transmission range to give an average of six neighbors for each node. Takagi and Kleinrock revised this result, and gave a new optimal nodal degree eight [69]. Other nodal degrees were also obtained under different transmission protocols and inhomogeneous transmission ranges [69–71].

This work either implicitly or explicitly assumed that the network is connected. For example, [68] considers only networks up to size 150, and concludes that an average degree of 4 is sufficient for connectivity. Among others, Philips *et al.* [33] have proved that with a constant average nodal degree, a sufficiently large network is almost surely disconnected, dispelling the notion of a “magic number” [68]. However, the work on maximising throughput remains interesting in that it demonstrates that, at least for small networks, it may be optimal to use a larger number of neighbours than the minimum required for connectivity.

## 6. Clustering, Virtual Backbone and Minimum Connected Dominating Set

Simply maintaining the connectivity of a WSN is not sufficient for data dissemination. Routing must also be considered. This section will consider connectivity issues arising in one popular approach: cluster-based routing.

Routing is a major challenge for WSNs. The large number of sensors involved means that efficient routing is required. However, their typical data rates are much lower than other large networks such as the internet. Hence the relative cost of the signalling overhead of standard routing algorithms such as distance vector routing [72] or link state routing [73] can be excessive. This is exacerbated by the fact that nodes have very little computational power, but each node typically has a very large number of neighbours because wireless is a broadcast medium. Moreover, the set of neighbours is rather nebulous because wireless links vary in their reliability. Using reliably, short hops causes an excessive number of transmissions and hence excessive interference; conversely, using longer hops risks the need for retransmissions. (This can be addressed by opportunistic scheduling [74, 75], which is out of scope of this paper.) Finally, unstructured routing protocols require all potential relays to have their radio circuitry powered all of the time, in case they are called upon to relay data. This is a significant overhead in the ultra-low-power designs envisioned for WSNs.

One common solution to these problems is to perform routing through a specific subset of nodes, called a virtual backbone, to which all other nodes connect in a single hop. A good virtual backbone can simplify the routing process and can reduce the overall network energy consumption in two ways. First, as only nodes in the virtual backbone forward packets, non-backbone nodes can spend more time in a low-power idle mode. Second, all sensor nodes need to perform in-network processing and data aggregation. Doing this within virtual backbone nodes can eliminate redundant data and relax the packet transmission burden, which leads to energy saving.

For a given WSN, we typically wish to find a virtual backbone with the minimum number of nodes, to maximise the potential energy saving. However, successful packet delivery requires that the nodes in the virtual backbone remain connected, and that every other node is within range of a backbone node.

In this section we first introduce cluster-based routing, which groups nodes according to their geometric positions, and selects a head node for each group (i.e., cluster). All the cluster heads form a virtual backbone. We also discuss a graph theoretic concept, Minimum Connected Dominating Sets (MCDS), which model a small connected relay set. We also cover the state-of-the-arts algorithms for MCDS construction.

### 6.1. Cluster Routing Protocols

Clustering is an effective way to achieve efficient routing in WSNs [4, 76]. It can reduce the communication overhead, and simplify routing and management operations in densely deployed WSNs. Under clustering routing, the whole WSN is partitioned into several clusters, each of which contains nodes in close proximity. In each cluster, there is a cluster head. Inter-cluster communications can only occur between cluster heads, hence nodes in different clusters must connect to their cluster heads first if they need to communicate with each other. Examples of cluster-based routing schemes include LEACH [77], HCR [78], CBRP [79], and CEDAR [80]. CEDAR and CBRP are general cluster-based routing algorithms for wireless ad hoc networks, while LEACH and its extension HCR are specialized for WSNs. Other cluster-based routing algorithms for WSNs include TEEN [81] and APTEEN [82].

The usage of cluster-based routing in WSNs is also motivated by the requirements of in-network processing and data aggregation to reduce energy consumption, as such operations can be spontaneously performed at cluster head nodes. It should be noted that once the clusters are formed in a WSN, traditional proactive routing schemes such as distance vector routing and link state routing, as well as reactive routing approaches such as DSR [83] and AODV [84] can be used among those cluster head nodes, either for routing information updates or for data delivery.

Cluster-based routing is a special example of backbone-based routing [85], if nodes in a backbone are considered as cluster heads. To emphasise that a backbone comprises selected wireless nodes rather than being a wired backbone of base stations, the former is often called a “virtual” backbone. A virtual backbone in a WSN is a set of nodes that are connected together, and routing operations are restricted to those nodes in the backbone. Therefore, to ensure its communications to other nodes, each sensor should connect to at least one node in the virtual backbone in a single hop, while virtual backbone nodes can communicate with each other via multi-hop paths. It is clear that the nodes in the virtual backbone need to maintain the connectivity among each others. A virtual backbone also has other functions, such as route maintenance, transmission scheduling, and broadcasting [86].

**Figure 2.**
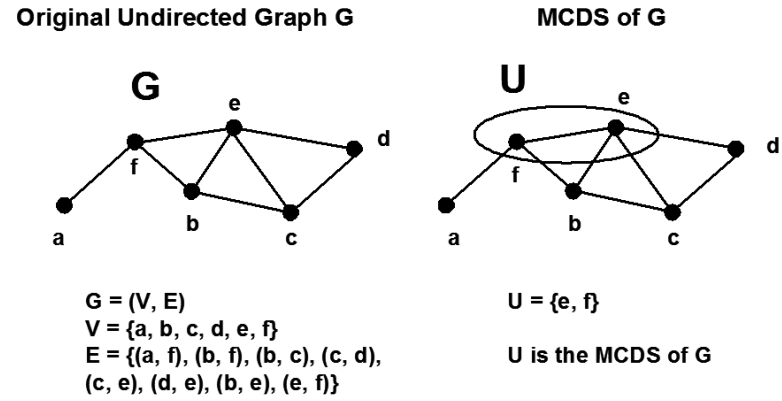
An example of Minimum Connected Dominating Set.

A virtual backbone of a WSN can be modelled by a **C**onnected **D**ominating **S**et (CDS) in the connectivity graph representing the WSN. For a graph *G* = (*V, E*), in which *V* is the set of vertices with cardinality *n*, and *E* is the set of edges, a dominating set (DS) is defined as a subset of *V*, such that any vertex in *V* is either an element of the DS or has at least one neighbour in the DS. If such a dominating set is connected, i.e., any arbitrary pair of vertices in the DS are connected by a path containing only elements of DS, then it is called a connected dominating set (CDS). A graph can have multiple DS and CDS. The CDS that has the smallest cardinality is named a **M**inimum **C**onnected **D**ominating **S**et (MCDS). Just like a CDS, an MCDS need not be unique. Figure 2 illustrates the concept of MCDS by a simple example. A network is modelled as a connected graph *G* = (*V, E*), in which *V* = {*a, b, c, d, e, f*} is the set of vertices, representing all the nodes in the network, while an edge between two vertices in the graph stands for the existence of a direct connection between the corresponding sensor nodes, and the edges set *E* = {(*a*,*f*), (*b, f*), (*b, c*), (*c, d*), (*c, e*), (*d, e*), (*b, e*)(*e, f*)} is the collection of all the connections. According to the previous definition, the MCDS of *G* in Figure 2 is the subset *U* = {*e, f*} ⊆ *V*. Since we model WSNs by graph models, a connected dominating set in the corresponding graph can form the virtual backbone for a WSN. Also, if we can find the MCDS in the graphs, we can construct a virtual backbone with the least number of nodes.

Unfortunately, finding an MCDS in a given connected graph is not as easy in practical situations as in the previous example. This problem is known to be NP-hard [87]. Some papers study properties of the MCDS directly, such as Li *et al.* [88], which evaluated the cardinality of the MCDS probabilistically for random networks. However, research activity on MCDS problem mainly focuses on providing heuristics or approximation algorithms, which achieve near MCDS construction.

Note the difference between heuristics and approximation algorithms. An algorithm *A* is called a *α*-approximation if it can be proved that *A* will not be more than a factor *α* times the optimum solution *s_opt_*, i.e., *A* ≤ *αs_opt_*. The *α* is called the approximation ratio of *A*. In contrast, a heuristic is any algorithm which appears to work well in most cases.

Existing algorithms for constructing an approximate MCDS can be classified into three categories: constructive, pruning-based and multipoint-relay-based algorithms. Constructive algorithms approximate the MCDS in a graph by gradually adding nodes to a candidate set. In contrast, pruning-based algorithms begin with taking a large candidate set, then detect and remove redundant nodes to eventually obtain a small CDS. The last type, multipoint-relay-based algorithms, allows each node to determine its smallest one-hop message relay set, and all nodes selected as relay nodes for a particular message relaying form a CDS.

For all three types of algorithms, the connectivity among the relay nodes must be considered. A common approach used by constructive algorithms is first to select a subset of a CDS, called a **M**aximal **I**ndependent **S**et (MIS), and then using some additional nodes to provide connectivity among nodes in the MIS. Algorithms in this class are generally differentiated according to the approaches used to produce the MIS and to find the additional nodes. An independent set (IS) of a graph *G* = (*V, E*) is defined as a subset *V̅* ⊆ *V*, such that there is no edge between any two vertices in *V̅*, i.e., ∀*u,v*∈*V̅*, (*u, v*) ∉ *E*. An independent set that is not a proper subset of any other independent set is called a maximal independent set. Note the difference between a maxi*mal* independent set and a maxi*mum* independent set. The latter is defined as an independent set with the largest cardinality, but there can be multiple maximal independent sets with varying cardinality. While construction of a maximal independent set can be solved in polynomial time by greedy algorithms, finding the maximum independent sets is NP-complete [87]. In contrast to constructive algorithms, pruning-based algorithms generate a large CDS first, and then remove redundant nodes while keeping the connectivity among the remaining nodes in the CDS. In terms of the multipoint-relay-based algorithms, the connectivity of the constructed dominating set is guaranteed in the selection of multipoint relay nodes.

These three types of algorithms are described in more detail in the following subsections. Typical examples of each type are also discussed.

### 6.2. Constructive Algorithms

Guha and Khuller's seminal paper [89] shows that a *β* approximation for the problem of finding the MCDS corresponds to a 2*β* approximation for another NP-hard problem, the travelling tourist problem. It uses this result as the basis for two polynomial time approximation algorithms for the MCDS problem. These two algorithms have approximation factors of 2(*H*(Δ) + 1) and *H*(*Δ*) + 2, respectively, in which Δ is the maximum nodal degree and *H* is the harmonic function [90].

The first algorithm approximates the MCDS by greedily creating a tree *T*. All the non-leaf nodes of *T* form the approximate MCDS, which is indeed a CDS. Nodes have three different states, which are black, grey and white. Black nodes are nodes in the final CDS, so called dominators. Grey nodes are candidates of dominators, and white nodes are those nodes not yet in *T*. Initially, all the nodes are white nodes, and the algorithm finds out the node with maximum degree of white neighbors, marks it black, and marks all its white neighbors grey. The algorithm then iteratively marks the grey node or the pair of connected grey nodes that have maximum white neighbors to black, and update those white neighbors to grey state. The set of black nodes in the final result is the CDS. Note the correspondence of grey and black nodes to candidate and confirmed nodes in Dijkstra's algorithm [91]; however a notable difference between the algorithms is that the order of processing the grey nodes is based on their connectivity rather than the cost of the path.

To develop the second algorithm, Guha and Khuller introduced a new concept called *piece*, defined as either a connected black component, or a white node. The second algorithm selects nodes with maximum degree of *pieces* as dominators until there are no white nodes. It then connects all the dominators by constructing a Steiner tree.

A distributed algorithm is called *localized* if each node decides on its own behavior based on only the information from nodes within a limited hop distance, e.g., one or two hops. With localized algorithms, topology changes in part of the network can be isolated, and may not affect the rest of the network. In contrast, a distributed non-localized algorithm requires nodes to acquire global knowledge of the network to perform correct operations [92].

Neither of the above algorithms is localized, as they need global information to select the nodes with maximum degree of white neighbors or *pieces*. Das and Bharghavan [93] proposed to construct a virtual backbone in a wireless ad hoc network by using its MCDS, and developed a distributed implementation of Guha and Khuller's algorithm. Das and Bharghavan's distributed algorithm is also non-localized, as the centralized algorithm it is based on finds a non-localized solution.

Another distributed heuristic to compute the MCDS for a wireless ad hoc network was proposed by Alzoubi and Wan [94]. The algorithm is non-localized as it involves the construction of a spanning tree. Indeed, this algorithm follows the same idea as the second algorithm of Guha and Khuller. It selects a dominating set first, and then connects nodes in the dominating set by choosing some additional nodes as connectors. The selection of the dominating set is based on the construction of a maximal independent set (MIS), and the connection step is completed by selecting some additional nodes to form a spanning tree. The authors provided performance bounds by applying the property that a node in a unit disk graph cannot have more than five independent neighbors [95]. If *n* is the number of vertices for the whole network, the algorithm has time complexity of *O*(*n*), and message complexity of *O*(*n* log(*n*)), while providing a constant approximation factor. This algorithm is non-localized as it needs a leader node to generate a rooted tree. To overcome this drawback, Alzoubi *et al.* [96] proposed another algorithm, which generates an MIS based on multiple leaders, and hence is localized. The algorithm obtains a CDS with cardinality at most *192*|*OPT*| + 48, in which |*OPT*| is for the cardinality of the MCDS. The main drawback of this algorithm is the low efficiency of the connector selection procedure. Other work based on computing and connecting a MIS include [97–99], and references therein.

### 6.3. Pruning-Based Algorithms

Besides constructive algorithms, there also exist other algorithms which are based on pruning procedures to approximate MCDS. As indicated by its name, a pruning-based algorithm gradually reduces a candidate CDS according to some greedy criteria, and the left-over set after the reduction is the approximate MCDS.

Wu and Li [100] proposed a two step localized algorithm to construct a CDS in a network. In the first step, each node in the network gets information of its two-hop neighbors via the exchange of beacons. Every node that has two neighbors that are not within the radio range *r* of each other marks itself as a dominator. The second step is a pruning process, which takes out some redundant nodes from the dominator set generated in the first step. There exist two rules to reduce the size of the CDS generated in the first step. A node *u* can be removed from the CDS if its closed neighborhood is a subset of that of another node *v* in the CDS, or the open neighborhood of *u* is a subset of the union of the open neighborhood of another two nodes *v* and *w*, which are in the CDS. The open neighborhood of a node *u* is defined as the set of all the nodes that direct link to *u*. The closed neighborhood of *u* is the union of its open neighborhood and *u*.

Wu *et al.* [101] extended this algorithm to make it energy aware. For an arbitrary node *u*, the remaining energy level of *u* is considered when the pruning process is performed. Nodes with lower energy level are preferentially removed.

Butenko *et al.* [102] propose another algorithm that uses pruning. This algorithm takes the entire set of nodes *V* as the initial candidate CDS, and then greedily removes nodes. In order to determine whether a node can be removed from the current CDS, the connectivity of the remaining nodes is tested. The node can be removed only if the remaining candidate CDS is still connected. This algorithm has high message complexity due to the operations of connectivity tests, especially for large dense networks. However, simulation results show that the cardinality of the CDS it finds is comparable to existing algorithms. Sanchis [103] also discusses a similar approach in the construction of dominating sets.

### 6.4. Multipoint Relaying (MPR) in CDS Construction

Multipoint relaying is a technique that is widely used for flooding in wireless ad hoc networks. In multipoint relaying, each node *u* in the network selects a subset of its first-hop neighbors, named the multipoint relay set (MRS), which are responsible for forwarding packets from node *u* to *u*'s two hop neighbors. Only nodes in the MRS of *u* can forward packets sent by *u*. Finding a minimum size MRS in a network is shown to be NP-Complete [104]. The MRS of a node *u* forms a local dominating set, which dominates nodes within two hops distance from *u*. Although the MRSs do not directly generate a global CDS, they can be extended to do so.

Note that in [104], node *u* and its MRS only form a local CDS. Some algorithms approximate MCDS for the whole network based on MRSs. One such example is MPR-CDS where a source-independent MPR algorithm is proposed to construct a global CDS based on the MPR approach [105]. The algorithm assumes that each node in a connected network has a unique ID. The global CDS is constructed based on the ID of nodes, according to the following criteria. A node *u* is added into the CDS if it has the smallest ID among all its first-hop neighbors, or it is a member of the MRS of its neighbor with the smallest ID. The time complexity is shown to be *O*(Δ^2^), where **Δ** is again the maximum node degree, while the message complexity is *O*(*n*).

However, as pointed out by Wu [106], the CDS generated by MPR-CDS can be inefficient as the nodes selected by the first criterion may not be necessary. This is because the first criterion only select CDS nodes based on their IDs. Hence for certain network topologies, nodes chosen by the first criterion are redundant. Therefore an *enhanced MPR* (EMPR) was proposed by modifying the MPR-CDS in both the selection of the smallest ID nodes and the original MPR selection. Due to these two modifications, the EMPR has high computation complexity.

Other MPR-based CDS construction heuristics include Chen *et al.* [107], Wu *et al.* [108, 109], and others. For a comprehensive survey about multipoint-relay-based broadcast schemes, see Ou *et al.* [110], which includes multipoint-relay-based CDS construction. Note that all the discussions of MCDS are based on the fact that the network is connected. When dealing with a disconnected network, we can only find CDSs of each connected part of the network.

As the MCDS problem is NP-complete, future research will likely to focus on providing heuristic algorithms and approximations that are efficient and scalable. Another possible new research direction is to focus on networks with specific structures, as done by Li *et al.* [88].

## 7. Placement of Nodes

The placement of nodes largely influences the operations and performance of WSNs, as sensor nodes must be able to observe events of interest, and transmit the information to data collection centres. Moreover, sensor placement also affects the resource management in WSNs [111]. Sensor networks often monitor specific points, e.g., monitoring intersections or animal nesting sites, which constrain the node placement. In such cases, the placement issue is highly application-specific. Our survey is only of cases where the aim is to monitor an entire area, either continuous or on a discrete grid.

According to the roles that the deployed nodes play, node placement can be classified into placement of ordinary nodes, and placement of relay nodes, respectively. The former focus on the deployment of normal sensors, while the latter places a special type of nodes, which are responsible for forwarding packets.

### 7.1. Placement of Ordinary Nodes

The foremost step required for a WSN to perform its designed functions is deploying all the sensor nodes to form a WSN. As mentioned previously, sensors can be placed exactly on carefully engineered positions, or thrown in bulk on random positions [3]. Each of these will be considered in turn.

Most work on deterministic placement seeks to determine the “optimal” placement pattern. Different optimality criteria are used, according to the applications and goals of the WSNs. A common objective is to minimise the number of required sensors needed, subject to the constraint that the whole sensing field is monitored by the deployed sensors. This is equivalent to finding the minimum number of nodes such that every position in the sensing field is within the sensing field of at least one node.

Minimising the number of sensors can take the form of an “art gallery” problem [112], which aims to find the minimum set of locations for security guards inside a polygonal art gallery, such that the boundary of the entire gallery is visible by at least one of the security guards. Gonzólez-Banos [113] proposed a randomized algorithm to solve the art gallery problem to find locations for sensor nodes.

However, in the art gallery problem, all security guards are assumed to have infinite vision if there is no obstacles. This assumption does not hold for WSNs in which sensor nodes have limited sensing ranges. It was shown that arranging sensors at the centres of regular hexagons is optimal for a WSN with a large sensing field, given that all the sensor nodes have identical limited sensing ranges [114]. This result has long been known in mathematics community; see Kershner [115]. There is also some recent work published discussing the node deployment scheme through the maximization of coverage, through techniques such as Virtual Force Algorithm and Tabu Search, see [116–118].

The above work on the node placement only considered the coverage constraint that the WSN needs to be able to observe any positions within the sensing field. They did not include the discussion about the connectivity, or they implicitly assumed that the WSN formed by the obtained pattern was connected, regardless the transmission ranges of sensor nodes. Nevertheless, this assumption may not be true. To find the optimal node placement pattern subject to both the coverage and connectivity constraints, Biagioni and Sasaki [119] modelled the problem as an optimization problem that minimizes the number of required sensor nodes, under the constraints that any position of the sensor field is under surveillance of at least one node and all nodes are connected. However, the paper did not find a general solution. Instead, it discussed a wide range of regular sensor network deployment patterns. These patterns include circular, star, and grid patterns, such as triangular, square and hexagonal grids.

Iyengar *et al.* [120] proved that a strip-based pattern is nearly optimal for this problem for large networks in two-dimensional space. The strip-based pattern, nodes are arranged along many parallel horizontal strips, and nodes in different horizontal strips can communicate via some nodes located as a vertical ribs. Bai *et al.* [121] extended the work in [120] by proving that this strip-based pattern is the asymptotic optimal pattern for an infinite network. A slight modification of the asymptotic optimal strip-based pattern, which is optimal to achieve coverage and 2-connectivity was also proposed in [121]. This modified pattern has two vertical ribs of nodes on either end of the sensing fields instead of a single vertical rib. Very recently, Bai *et al.* [122] published new results, which proposed optimal sensor placement patterns to achieve coverage and *k*-connectivity (*k* ≤ 6). Note that *k*- connectivity can provide fault tolerance. Above work assumed that the WSN was deployed in an open area, without obstacles such as buildings or stationary. In contrast, Wang *et al.* [123] developed a general sensor deployment scheme, which can efficiently determine sensor positions for an arbitrary sensing field, probably with obstacles.

Studies on objectives other than minimising the total number of sensors are also available. Khan [124] studied the placement of a given number *n* of nodes in a grid sensor field. The aim was to minimise the distance between sensors for fault tolerance reasons, subject to the constraints that all the *n* sensors are placed on grid points, and all the grid points are sensed by some sensors. Other work adopting grid-based deployment include Chakrabarty *et al.* [125], and Lin *et al.* [126]. Chakrabarty *et al.* [125] tried to find the node placement scheme that minimizes the cost of sensors to meet the complete coverage constraints. In particular, Lin *et al.* [126] developed an algorithm to solve the sensor placement problem on a grid field. Each grid point was coded according to the coverage relationship to sensors. The algorithm aimed to find the set of grid points that can minimise the maximum coding distance between any pair of grid points, subject to the cost limitation.

The above mentioned work [114, 115, 119–126] was based on deterministic sensor placement. However, for large scale WSNs, random placement has also received much attention. Ishizuka and Aida [127] proposed three types of random placement models. The authors studied the fault-tolerance and sensing coverage through simulations, and suggested that the placement model which assumes that sensors are uniformly distributed in terms of the network radius and angular direction from the sensor field centre is the best candidate for random node placement. Others (see [128] for an example) assume that all nodes follow identical Gaussian distribution.

Models of random placement usually assume that nodes are independently and uniformly distributed in space. It is not clear that this is a suitable model, although its tractability is appealing. Moreover, it has been shown that several models of random motion of nodes eventually yield a uniform distribution of nodes. Blough *et al.* [129] showed by simulation that a uniform distribution results if the initial distribution of nodes is uniform distribution, all the nodes move according to the random waypoint model and all the nodes move for a large number of steps of Brownian-like motion, which represents unintentional movement. Miller [128] showed that a uniform distribution results even if the initial distribution was Gaussian instead of uniform. Note however that these mobility models are no less arbitrary than the initial assumption of uniform placement. There is a dearth of literature on the actual distributions yielded by the proposed methods of random deployment.

### 7.2. Placement of Relay Nodes

In a WSN, if a small set of special nodes, whose main function is packet forwarding, are deployed, the management and network operations in the WSN can be potentially simplified drastically. These nodes are called relay nodes, and have attracted flourishing interests [130–134]. A fundamental problem which arises when establishing such a network is where to deploy those relay nodes to achieve the required grade of service, while meeting the system constraints. That is the topic of this subsection.

The problem of relay node placement can be categorized into either single-tiered or two-tiered, according to the data forwarding scheme adopted by the WSN. If both of relay nodes and ordinary sensor nodes can forward packets, this is single-tiered relay node placement. In contrast, in a WSN with two-tiered relay node placement, only relay nodes can forward packets. In a two-tiered system, the relays form a virtual backbone, and must be a connected dominating set (CDS) of the WSN.

We survey the prior work related to these two types of relay placement. In the following discussion, the transmission ranges for relays and ordinary sensors are denoted *R* and *r*, respectively.

Cheng *et al.* [130] developed algorithms to place the minimum number of relay nodes and maintain the connectivity of a single-tiered WSN, under the assumption that *R* = *r*, i.e., all relays and ordinary sensors have identical transmission ranges. The problem was modelled by the *Steiner Minimum Tree with Minimum Number of Steiner Points and Bounded Edge Length (SMT-MSP)* problem, which arose in the study of amplifier deployment in optical networks, and was proved to be NP-hard [135]. Based on a minimum spanning tree, Lin and Xue [135] developed an algorithm to solve the SMT- MST problem. They proved it to have an approximation ratio of 5, which Chen *et al.* [136] tightened to 4. In the same paper, a 3-approximation algorithm was also proposed. Based on Lin and Xue's algorithm, Cheng *et al.* [130] proposed a different 3-approximation algorithm and a randomized 2.5-approximation algorithm.

In order to provide fault-tolerance, Kashyap *et al.* [137] studied how to place minimum number of relays such that the resulted WSN is 2-connected, when relay nodes and ordinary sensor nodes have identical transmission range, i.e., *R* = *r*. A constant approximation algorithms with time complexity *O*((*kn*)^2^) was developed. Recently, Zhang *et al.* [134] improved the results of Kashyap *et al.* by developing algorithms to compute the optimal node placement for networks to achieve 2-connectivity, under the more general condition that *R* ≥ *r*. These algorithms aimed to minimise the number of relay nodes while providing fault-tolerance.

The setting that only relay nodes can perform the packet forwarding is known as the two-tiered infrastructure. Pan *et al.* [132] first investigated the two-tiered infrastructure for optimal node placement. Further studies considering an i.i.d. uniformly distributed sensor location with *R* ≥ 4*r* were given in [131, 133]. Based on [130] and [131], Lloyd and Xue [138] developed algorithms to find optimal placement of relay nodes for the more general relationship *R* ≥ *r*, under single-tiered and two-tiered infrastructures.

Apart from the above unconstrained relay node placement, in which relay nodes can be placed on anywhere, recent work has started to investigate constrained relay node placement problem which captures the practical consideration such as interferences or forbidden regions prevent relay nodes from being placed on certain positions. Some recent work investigating constrained relay node placement problem is given in [139, 140].

## 8. Concluding Remarks

Wireless Sensor Networks have the potential to revolutionize our everyday life, as they provide a flexible approach for us to observe the surrounding environment, and respond to events. The availability of tiny battery-powered sensor nodes, embedded with sensing, processing, and communication capabilities, which are wirelessly networked together via multi-hop communication, increase the opportunities for WSNs to find applications in a wide range of areas. Along with the opportunities, there are also challenges and requirements for the successful deployment and operations of WSNs. This survey has focused on the implications of the need for connectivity.

Ensuring connectivity of a WSN is challenging when sensors have random locations, either because of mobility or initial random deployment. A substantial body of literature has been written on this problem, including deep theoretical results applied to simple models of i.i.d. uniformly distributed nodes with circular radio footprints. This model is widely accepted as it is analytically tractable. An important open research problem is to generalise these results to more realistic propagation models, including effects such as shadowing and non-uniform distribution of nodes, and to determine what engineering insights can be drawn from the theoretical asymptotic results.

Connected subsets of nodes also play an important role in WSNs. As we have seen, cluster routing uses a connected “backbone” of nodes to simplify routing, and minimise the work required from the majority of sensor nodes. Finding the smallest such backbone is equivalent to finding a Minimum Con-nected Dominating Set in the corresponding graph, which is known to be NP-complete. Further research is needed both into finding more efficient, more accurate or simpler suboptimal solutions to the MCDS problem, and also into the benefits which can be obtained by using non-minimum backbones. Such benefits include reduced path lengths, and increased resilience. Such research will play an important part in bringing about the benefits that sensor networks have to offer.
